# Supplementary material for: Analysis and comparison of the trends in the burden of motor neuron disease in China and worldwide from 1990 to 2021
Source: PLoS One. 2026 Feb 26;21(2):e0343818. doi: 10.1371/journal.pone.0343818 (PMC12944794; doi:10.1371/journal.pone.0343818)
Supplement: Attachment — Submitted filename: Response to Reviewers.docx [file pone.0343818.s002.docx]

Manuscript ID : [PONE-D-25-27273]

Title: [Analysis and comparison of the trends in burden of motor neuron disease in China and worldwide from 1990 to 2021]

****Dear editor and Reviewers,****

****We wish to express our sincere gratitude to you and the reviewers for the valuable time and constructive comments provided on our manuscript. The insightful suggestions have been instrumental in helping us significantly improve the quality of our work.We have carefully considered all points raised and have revised the manuscript accordingly. Below, we provide a point-by-point response to each comment.The changes in the manuscript have been highlighted in the “Revised Manuscript with Track ” file for your convenience. We believe that the manuscript has been strengthened considerably and now fully meets the publication standards of** PLOS ONE**.****

****Response to Academic Editor’s Comments:****

****We have ensured that our manuscript now fully complies with PLOS ONE’s style requirements, including proper file naming. The corresponding author’s ORCID iD has been validated in the submission system. The abstract in the manuscript and the online submission form are identical. We have evaluated the literature suggested by the reviewers and cited relevant works where appropriate.****

****Response to Reviewer #1****

****We thank Reviewer #1 for their thorough review and positive feedback, as well as for the highly constructive suggestions to enhance our manuscript.****

****Comment 1: Discussion of Mechanisms: Consider expanding your discussion on the potential biological, environmental, or healthcare-related factors that may influence the observed trends in MND burden.****

****Response: We sincerely thank the reviewer for this excellent suggestion. We have now substantially expanded the 'Discussion' section to include a dedicated analysis of potential drivers. We now explore biological (e.g., genetic factors like C9ORF72), environmental (e.g., potential role of environmental toxins), and healthcare-related (e.g., improvements in diagnostic capacity, aging population) factors that may underpin the trends identified in our study.****

****Comment 2: Contextualization: A more detailed comparison with specific countries or regions, especially those with higher MND burdens, would enrich the context of your findings and enhance their applicability.****

****Response: As suggested, we have enriched the context of our findings by adding detailed comparisons with specific high-burden countries/regions identified in the GBD study . We discuss similarities and differences in trends, and hypothesize on the potential reasons (e.g., genetic backgrounds, healthcare systems, environmental factors) for these disparities.****

****Comment 3: Literature Review: Expanding your literature review to include more recent studies related to MND could better situate your work within the existing body of knowledge and highlight its relevance.****

****Response: We have updated the 'Introduction' and 'Discussion' sections to incorporate several recent studies (published within the last 3-5 years) on MND epidemiology and risk factors, which better situates our work within the current scientific conversation and highlights its timeliness.****

****Comment 4: Integration of Figures and Tables: Improving the integration of figures and tables into the narrative can facilitate reader understanding. Providing more detailed descriptions of each figure/table in the main text would be beneficial.****

****Response: We have carefully reviewed the narrative flow and now provide more detailed descriptions and interpretations of each figure and table in the main text. For instance, when presenting key results, we explicitly guide the reader to the relevant figure/table and summarize the most critical takeaways.****

****Comment 5: Ethics Statement: Clarifying how data privacy and ethical standards were maintained, given the sensitive nature of health data, would strengthen your ethics statement.****

****Response: We have strengthened the ethics statement in the 'Methods' section to explicitly state that this study utilized fully anonymized and aggregated data from the GBD database, which is compliant with ethical standards and does not involve individual patient data, thus ensuring data privacy.****

****Comment 6: Conclusions and Recommendations: Strengthening your conclusions with specific recommendations for future research or public health interventions based on your findings could provide practical applications for your study****

****Response: We have thoroughly revised the 'Conclusion' section to move beyond a simple summary. We now provide specific, actionable recommendations for future research (e.g., conducting gene-environment interaction studies in China) and for public health policy (e.g., enhancing specialized neurology care in regions with rising burden).****

****Comment 7: Language and Clarity: A thorough proofreading of the manuscript is recommended to address any awkward phrasing or grammatical errors, thereby enhancing the clarity and readability of your work.****

****Response: We have undertaken a thorough proofreading of the entire manuscript, and it has been professionally edited to correct grammatical errors and improve phrasing, clarity, and overall readability.****

****Response to Reviewer #2****

****We thank Reviewer #2 for their critical and valuable comments, which have been crucial for helping us substantially strengthen the rigor and clarity of our manuscript.****

****Comment 1: Numerous grammatical errors, awkward phrasing, and poor flow. The manuscript needs major language editing for clarity and professionalism.****

****Response: We fully acknowledge this critical shortcoming and sincerely apologize for the language issues in the original submission. The entire manuscript has now undergone extensive language editing by a professional academic editing service. We have rewritten awkward phrases, corrected all grammatical errors, and improved the overall flow and professionalism of the text throughout.****

****Comment 2: Some results are inconsistently described (e.g., prevalence trends in China are reported as both increasing and decreasing at different points)****

****Response: We apologize for this critical oversight and inconsistency. We have meticulously re-examined the entire 'Results' section to ensure all descriptions are accurate and consistent. The specific example mentioned (prevalence trends in China) has been corrected and is now uniformly described throughout the manuscript as an increasing trend. We thank the reviewer for catching this error. (Please see Page 2,high Track Changes )****

****Comment 3: The discussion mainly repeats results instead of providing deeper insights or justification of the results from secondary data source (e.g., why Chinese mortality trends differ from global ones)****

****Response: This is a pivotal comment. We have completely restructured and rewritten the 'Discussion' section. We have removed the mere repetition of results and instead focused on providing deeper interpretation. We now explicitly hypothesize and justify why Chinese MND mortality trends may differ from global patterns, discussing factors such as the unique demographic transition, regional variations in healthcare infrastructure, and potential under-reporting in earlier years. (Please see the entirely revised 'Discussion' section )****

****Comment 4: Heavy reliance on GBD estimates, but the limitations of such secondary data (underdiagnosis, reporting bias, quality of Chinese epidemiological records) are acknowledged only briefly!!****

****Response: We agree entirely with the reviewer that a more candid discussion of the limitations is crucial. We have now expanded the 'Limitations' subsection within the 'Discussion' section to dedicate substantial space to this issue. We discuss in detail the potential impacts of underdiagnosis, reporting bias, and the quality of source data from China, and how these limitations might influence the interpretation of our trends.****

****Comment 5: Figures are acceptable but overloaded with information; some redundancy exists between text and visuals****

****Response: We have revised all figures to reduce information overload and improve clarity. We also ensured that the text does not merely repeat what is visually evident in the figures but rather interprets and highlights the key messages from them.****
