# Supplementary material for: Analysis and comparison of the trends in the burden of motor neuron disease in China and worldwide from 1990 to 2021
Source: PLoS One. 2026 Feb 26;21(2):e0343818. doi: 10.1371/journal.pone.0343818 (PMC12944794; doi:10.1371/journal.pone.0343818)
Supplement: Attachment — Submitted filename: Response_to_Reviewers_auresp_2.docx [file pone.0343818.s003.docx]

Manuscript ID : [PONE-D-25-27273R1]

Title: [Analysis and comparison of the trends in burden of motor neuron disease in China and worldwide from 1990 to 2021]

****Dear editor and Reviewers,****

****We wish to express our sincere gratitude to you and the reviewers for the valuable time and constructive comments provided on our manuscript. The insightful suggestions have been instrumental in helping us significantly improve the quality of our work.We have carefully considered all points raised and have revised the manuscript accordingly. Below, we provide a point-by-point response to each comment.The changes in the manuscript have been highlighted in the “Revised Manuscript with Track ” file for your convenience. We believe that the manuscript has been strengthened considerably and now fully meets the publication standards of** PLOS ONE**.****

1. ****Comment on Discussion Quality****

**Reviewer: Although expanded, the Discussion section continues to be largely descriptive and lacks sufficient analytical depth. Stronger interpretation of the trends, integration of recent findings, and clearer links to the presented data are needed.**

****Response: We agree with this critique. In the revised manuscript, we have completely restructured the Discussion section to move beyond description. We now provide a stronger analytical interpretation of the observed trends in ASIR, ASPR, ASDR, and mortality, explicitly linking them to potential drivers such as demographic shifts, healthcare policies, and environmental factors. We have integrated key recent findings to contextualize our results and have added dedicated paragraphs that directly discuss the implications of our data figures, avoiding mere repetition of results.****

2. ****Comment on Limitations****

**Reviewer: The Limitations section has been expanded, but the content remains too general. Please provide a more detailed, data‑specific discussion of how underdiagnosis, reporting bias, and GBD modeling choices may affect ASIR, ASPR, ASDR, and mortality trends.**

****Response: Thank you for this suggestion. We have significantly expanded the Limitations section to provide a data-specific and granular analysis. We now discuss in detail: (a) how potential underdiagnosis in certain regions may artificially lower ASIR and ASPR estimates (b) the possible impact of temporal changes in reporting standards on mortality and ASDR trends; and (c) specific GBD modeling choices and how they might introduce uncertainty into our trend analyses for each major metric.****

3. ****Comment on Literature Update****

**Reviewer: Only a minimal amount of new literature has been incorporated. Recent region‑specific and China‑specific studies should be critically reviewed and integrated into both the Introduction and Discussion.**

****Response: We apologize for this oversight. In this revision, we conducted a comprehensive literature search, with a focus on high-quality studies from China and the broader Asia-Pacific region. These have been critically integrated into the Introduction to better establish the research gap and into the Discussion to compare and contrast our findings with current regional evidence.****

****4. Comment on Methods Clarity****

**Reviewer: The Methods section still lacks essential detail. Please clarify GBD data sources, modeling framework, uncertainty intervals, age‑group structures, and Joinpoint analytical specifications.**

****Response: The Methods section has been comprehensively revised for clarity and completeness. We now provide: a detailed table listing all primary GBD data sources used; a clearer description of the GBD modeling framework; an explanation of how Uncertainty Intervals are calculated and interpreted; the specific age-group structure adopted; and the full specifications of the Joinpoint regression analysis.****

5. ****Comment on Figures and Narrative Integration****

**Reviewer: The manuscript continues to provide limited interpretation of figures. The text should explicitly reference figures and highlight the implications of visual data rather than repeating numerical patterns.**

****Response: We have systematically revised the narrative in the Results and Discussion sections to strengthen the integration with figures. Each figure is now explicitly referenced at the point of discussion. The text focuses on interpreting the visual trends, highlighting key turning points, geographical disparities, and their potential public health or policy implications, rather than reiterating numerical values already present in the figures or tables.****

6. ****Comment on Conclusions and Recommendations****

**Reviewer: The conclusion remains general. Please provide clearer and more actionable research and policy recommendations tied directly to your findings.**

****Response: The Conclusion section has been entirely rewritten. It now begins with a concise summary of the core findings directly derived from our analysis. Subsequently, we provided specific, actionable research recommendations, all of which are explicitly linked to the data and figures we have presented.****

7. ****Comment on Language and Style****

**Reviewer: Despite claims of professional editing, numerous issues with clarity, structure, and phrasing persist. A more rigorous language revision is required to ensure precision and readability.**

****Response: We place great emphasis on this comment. The manuscript has now undergone an additional round of professional polishing by several native English editors specializing in the editing of scientific publications (proof attached). This round of revisions focuses on enhancing sentence clarity, logical flow, grammatical accuracy, and overall readability, while ensuring scientific precision.****
